# Estimation of Vertical Ground Reaction Force during Single-leg Landing Using Two-dimensional Video Images and Pose Estimation Artificial Intelligence

**DOI:** 10.1298/ptr.E10276

**Published:** 2024-02-26

**Authors:** Tomoya ISHIDA, Takumi INO, Yoshiki YAMAKAWA, Naofumi WADA, Yuta KOSHINO, Mina SAMUKAWA, Satoshi KASAHARA, Harukazu TOHYAMA

**Affiliations:** ^1^Faculty of Health Sciences, Hokkaido University, Japan; ^2^Faculty of Health Sciences, Hokkaido University of Science, Japan; ^3^Faculty of Engineering, Hokkaido University of Science, Japan

**Keywords:** Acceleration, Anterior cruciate ligament, Biomechanical loads, Impact forces

## Abstract

Objective: Assessment of the vertical ground reaction force (VGRF) during landing tasks is crucial for physical therapy in sports. The purpose of this study was to determine whether the VGRF during a single-leg landing can be estimated from a two-dimensional (2D) video image and pose estimation artificial intelligence (AI). Methods: Eighteen healthy male participants (age: 23.0 ± 1.6 years) performed a single-leg landing task from a 30-cm height. The VGRF was measured using a force plate and estimated using center of mass (COM) position data from a 2D video image with pose estimation AI (2D-AI) and three-dimensional optical motion capture (3D-Mocap). The measured and estimated peak VGRFs were compared using a paired *t*-test and Pearson’s correlation coefficient. The absolute errors of the peak VGRF were also compared between the two estimations. Results: No significant difference in the peak VGRF was found between the force plate measured VGRF and the 2D-AI or 3D-Mocap estimated VGRF (force plate: 3.37 ± 0.42 body weight [BW], 2D-AI: 3.32 ± 0.42 BW, 3D-Mocap: 3.50 ± 0.42 BW). There was no significant difference in the absolute error of the peak VGRF between the 2D-AI and 3D-Mocap estimations (2D-AI: 0.20 ± 0.16 BW, 3D-Mocap: 0.13 ± 0.09 BW, *P* = 0.163). The measured peak VGRF was significantly correlated with the estimated peak by 2D-AI (*R* = 0.835, *P* <0.001). Conclusion: The results of this study indicate that peak VGRF estimation using 2D video images and pose estimation AI is useful for the clinical assessment of single-leg landing.

## Introduction

Anterior cruciate ligament (ACL) injury is a severe sports injury requiring surgical reconstruction followed by 6 to 12 months of rehabilitation to return to sports^1)^. In addition, a second ACL injury (i.e., graft tear or contralateral ACL tear) occurs in 15% of patients^2)^, and the reinjury rate in young patients returning to sports can be as high as 30%^3)^. Insufficient recovery of knee function has been considered a possible risk factor for a second ACL injury^4–6)^.

A large vertical ground reaction force (VGRF) during landing is a predictor of ACL injury^7,8)^. The ACL strain peaks at the same time as the VGRF during landing^9)^. Moreover, a cadaveric simulation study showed that axial tibiofemoral compression force leads to posterior femur displacement due to the posterior slope of the tibial plateau, which results in ACL injury^10)^. Additionally, patients who have undergone ACL reconstruction tend to demonstrate smaller VGRFs during single-leg landing on the surgical limb than on the contralateral limb, which is considered a compensatory strategy and may be associated with insufficient recovery of knee function^11)^. In fact, side-to-side asymmetry in the peak VGRF during single-leg landing is less pronounced in patients who can return to sports at the preinjury level than in those who can not^12)^. Furthermore, asymmetry in the knee extensor moment during landing is a predictor of a second ACL injury^13)^, which is associated with asymmetry in VGRF^14,15)^. Assessment of the VGRF during landing is crucial for patients following ACL reconstruction.

VGRF is typically measured using a force plate, the use of which is mostly limited to laboratory settings. Ideally, VGRF should be measured during actual sport performance. Therefore, researchers have explored alternative methods to estimate VGRF in clinical and sports settings. Estimation using kinematic data, i.e., the acceleration of the center of mass (COM), is one of those methods^16–20)^. Previous studies used a three-dimensional (3D) optical motion capture system to calculate the COM position and acceleration^16–20)^. However, this approach has limitations involving the requirements for a well-trained examiner, limited recording space, and a time-consuming measurement and analysis process. Consequently, it has not been applied in clinical and sports settings.

Recently, significant advancements have been made in pose estimation artificial intelligence (AI). OpenPose, an open-source pose estimation AI^21)^, can detect key body points such as joints from a two-dimensional (2D) video footage. This technology has been used to estimate joint angles and COM positions^22–26)^. Pose estimation AI combined with 2D video footage has the potential to overcome the limitations of 3D motion capture, including the need for a well-trained examiner, a recording space, and time-consuming measurement and analysis processes. If the VGRF can be estimated from a 2D video image using pose estimation AI, this modality can be widely applied to motion assessment in clinical and sports fields. Therefore, the purpose of this study was to determine the validity of VGRF estimation during a single-leg landing using a 2D video footage and pose estimation AI. We assessed the linear relationship between the estimated and the measured VGRF and compared the estimation errors with those obtained from an optical 3D motion capture system.

## Methods

### Participants

Eighteen young male participants (age 23.0 [1.^6^] years, height 173.5 [5.^7^] cm, and body mass 63.0 [8.3 kg]) were enrolled. Exclusion criteria were a history of lower extremity surgery, obvious deformities of the lower extremity, knee pain during the single-leg landing, and other conditions that could interfere with single-leg landing. Written informed consent was obtained from each participant before participation. This study was approved by the Institutional Review Board of Faculty of Health Sciences, Hokkaido University (approval number: 22-89).

### Procedures and data collection

The participants wore a uniform tight T-shirt and spats and were barefoot during data collection. Following a five-minute warm-up using a bicycle ergometer at a self-selected pace, a total of 50 retroreflective markers were attached to the participants. Marker placements on the pelvis and lower extremities were the iliac crest, anterior and posterior superior iliac spines, medial/lateral femoral epicondyles, medial/lateral malleoli, second metatarsal head/base, fifth metatarsal head, heel, and thigh/shank marker clusters^27)^. In addition, head, arm, and trunk markers were attached on the head, sternum, shoulder, elbow, and wrist. Then, the participants performed a single-leg landing task. The single-leg landing task is widely used for biomechanical assessment after ACL reconstruction^11)^. The participants stood on a 30-cm high box solely with their right leg and with their arms crossed over their chest. They were asked to drop off the box, land on a force plate with their right leg, maintain a single-leg stance for 5 s after landing, and look forward during the task. Practice trials were allowed for the participants to become familiar with the landing task.

The landing tasks were recorded using a commercial digital video camera (GoPro HERO11; GoPro, San Mateo, CA, USA), a 3D motion capture system (Cortex version 5.0.1; Motion Analysis, Santa Rosa, CA, USA) with seven infrared cameras (Hawk cameras; Motion Analysis) and a force plate (Type 9286; Kistler AG, Winterthur, Switzerland). The video camera recorded the front view at a height of 0.8 m and at 3 m away from the landing point. The pixels of the GoPro camera were set to 2704 × 1520 (2.7K), and a linear digital lens was used with horizon leveling. No additional distortion correction was applied in post-processing. The sampling rates were set at 240, 200, and 1000 Hz for the digital video camera, 3D marker trajectories, and force plate data, respectively. Three successful trials of the landing task were collected.

### Data processing

**Fig. 1.**
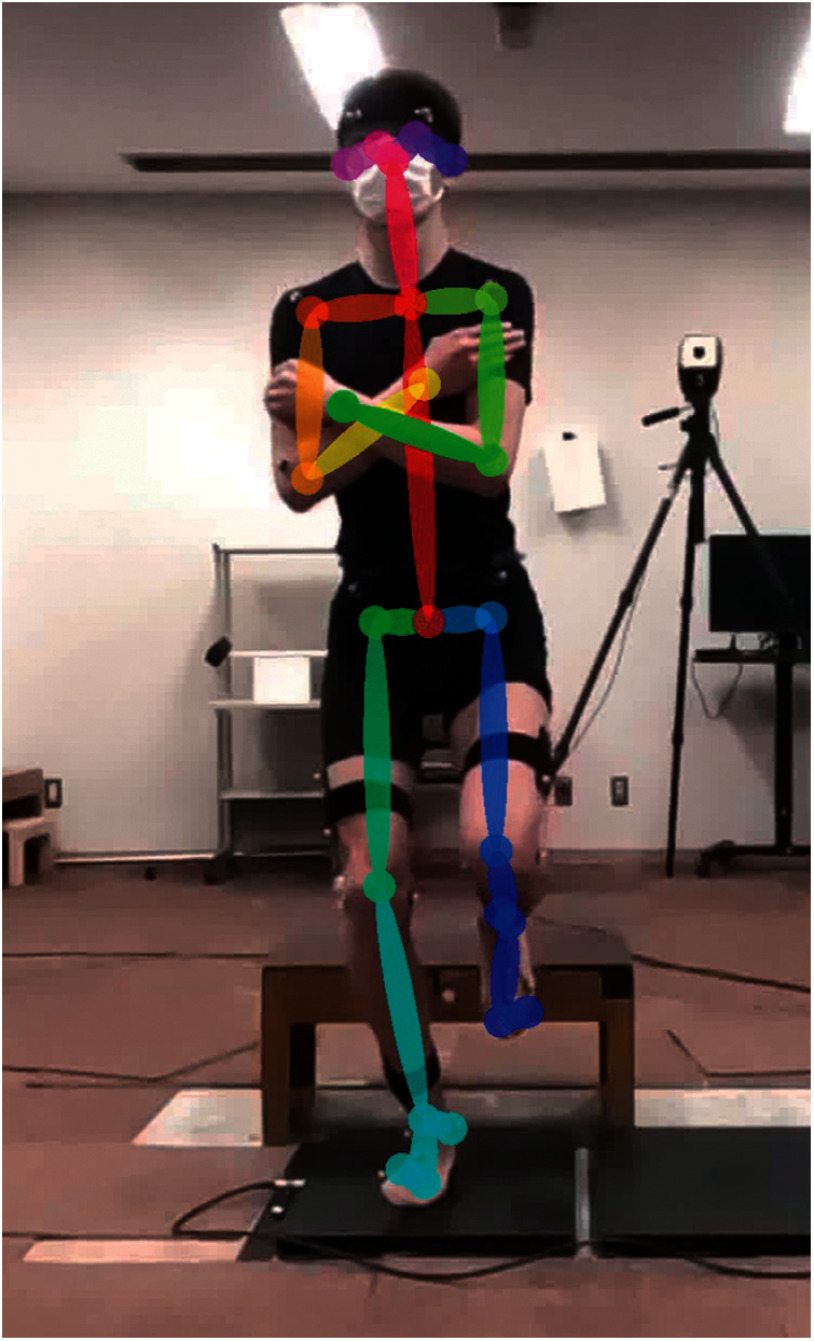
Estimation of key body points using a pose estimation artificial intelligence “OpenPose”


VGRF was estimated from the 2D video image with pose estimation (2D-AI) and 3D marker trajectories (3D-Mocap). The video image was analyzed using an open-source 2D pose estimation AI (OpenPose 1.7.0^21)^). OpenPose was run on a GPU (GEFORCE RTX 3700; NVIDIA Corp., Santa Clara, CA, USA). Analysis of 5 s of video took approximately 90 s. Our developed software (HUS motion analyzer, Hokkaido University of Science, Sapporo, Japan) provides a user interface to run OpenPose and enables the user to set the origin of 2D coordinates on the image, convert units from pixel to meter, and output them to a CSV file. In the present study, the units were converted using a 30-cm platform as a reference. OpenPose can detect 25 key body points from a 2D video image (Fig. 1), which include the nose, the neck, the midpoint of the hip joints, bilateral eyes, ears, shoulder joints, elbow joints, wrist joints, hip joints, knee joints, ankle joints, 1st and 5th toes, and heels. The coordination of each body point was calibrated by the 30-cm height box on the video image. The following procedure was performed using MATLAB (MathWorks, Inc., Natick, MA, USA). A fourth-order Butterworth lowpass filter was adopted for each body point. The cut-off frequency of the lowpass filter was verified from 6 to 12 Hz with 1 Hz intervals because the optimal cut-off frequency has not been established. The COM position was calculated for each segment from the filtered coordinates of key points according to previously reported anthropometric data^28)^. Then, the vertical acceleration of the COM (*a*_COM_) was calculated by second-order differentiation of the vertical COM position, and the VGRF was estimated from Newton’s equation of motion (Eq. (1))^17–19)^.

(1) $$\text{Estimated VGRF}=m(a_{\text{COM}}+g)$$

where *m* is body mass, and *g* is gravitational acceleration.

3D marker trajectories were low-pass filtered using a fourth-order, zero-lag Butterworth filter with a 12-Hz cut-off frequency^29)^. Then, similar to the 2D-AI estimation, the COM position and acceleration were calculated^28)^, and the VGRF was estimated from Newton’s equation of motion (Eq. (1))^17–19)^. VGRF data from measurements using the force plate were filtered using a fourth-order Butterworth filter with a cut-off frequency of 15 Hz^29)^.

Both the measured VGRF and the estimated VGRF were derived during 150 ms after the initial contact (IC), and the peak VGRF during this phase and the time to peak VGRF from IC were derived. The analysis period was set to be approximately double the time between IC and the peak VGRF^30,31)^ because ACL injury is assumed to occur within this period^32)^. Then, both sets of VGRF data were time-normalized to 101 data points for comparison. IC was defined as when the VGRF first exceeded 10 N. The VGRF was also normalized to the body weight (BW).

### Statistical analysis

Data were presented as the mean (standard deviation [SD]). The estimated error of the peak VGRF was assessed using the absolute error (AE). The root mean square error (RMSE) was also calculated during the analyzed phase (150 ms after IC). The AE and RMSE were used to determine the optimal cut-off frequency of the lowpass filter for key body point coordinates estimated using a pose estimation AI. A linear relationship between the measured and estimated peak VGRF was determined using Pearson’s correlation coefficient and interpreted as *R* ≥0.9, extremely large; 0.7 ≤*R* <0.9, very large; 0.5 ≤*R* <0.7, large; 0.3 ≤*R* <0.5, moderate; 0.1 ≤*R* <0.3, small and *R* <0.1, trivial^33)^. Bland–Altman analysis was also performed. A paired *t*-test was used to examine the fixed error between the measured and estimated peak VGRF. The proportional error was examined using Pearson’s correlation coefficient. Additionally, intra-tester reliability was assessed using the intraclass correlation coefficient (ICC). The ICCs were interpreted as follows^34)^: ICC ≥0.75, excellent; 0.4 ≤ICC <0.75, fair to good; and ICC <0.4, poor. The statistical significance level was set at *P* <0.05. These statistical analyses were performed using IBM SPSS Statistics software (version 22; IBM Corporation, Armonk, NY, USA).

## Results

**Fig. 2.**
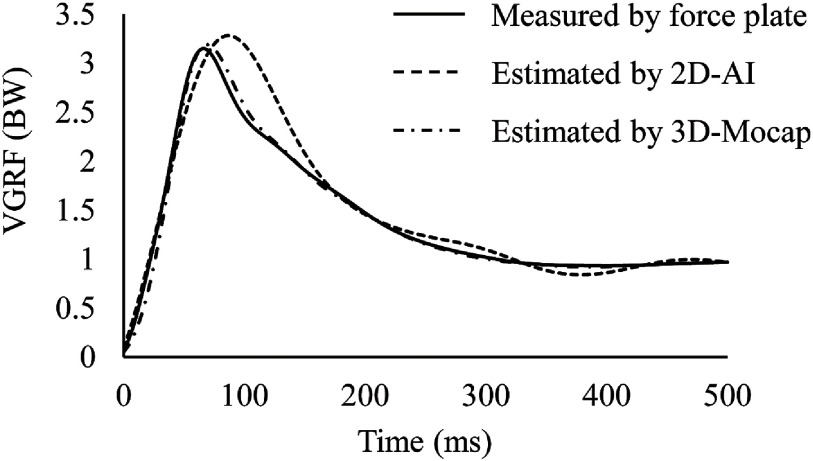
Average time-curves of VGRF VGRF, vertical ground reaction force; BW, body weight; 2D, two-dimensional video image; AI, artificial intelligence; 3D-Mocap, three-dimensional optical motion capture

Table 1 shows a comparison of the AE of the peak VGRF and RMSE for the analyzed phase among the tested cut-off frequencies of the lowpass filter for body point coordinates estimated by a pose estimation AI. The cut-off frequency of 7 Hz presented superior results with a minimal AE and RMSE compared to those of the other cut-off frequencies. Consequently, the results of 2D-AI lowpass filtered at 7 Hz were used for subsequent statistical analysis (Fig. 2).

**Table 1.** Comparisons of the errors among the tested cut-off frequencies of the lowpass filter for key body point coordinates estimated by a pose estimation

Frequency (Hz)	AE of the peak VGRF		RMSE during the analyzed phase	
	Distribution of trials with the lowest error [n (%)]	Mean (SD) [BW]	Distribution of trials with the lowest error [n (%)]	Mean (SD) [BW]
6	18 (33%)	0.26 (0.16)	11 (20%)	0.57 (0.16)
7	22 (41%)	0.20 (0.16)	14 (26%)	0.50 (0.12)
8	10 (19%)	0.24 (0.14)	16 (30%)	0.49 (0.12)
9	0	0.61 (0.23)	1 (2%)	0.86 (0.17)
10	4 (7%)	0.41 (0.19)	9 (17%)	0.64 (0.20)
11	0	0.49 (0.23)	3 (6%)	0.74 (0.18)
12	0	0.61 (0.23)	0	0.86 (0.17)

AE, absolute error; VGRF, vertical ground reaction force; RMSE, root mean square error; SD, standard deviation; BW, body weight

**Fig. 3.**
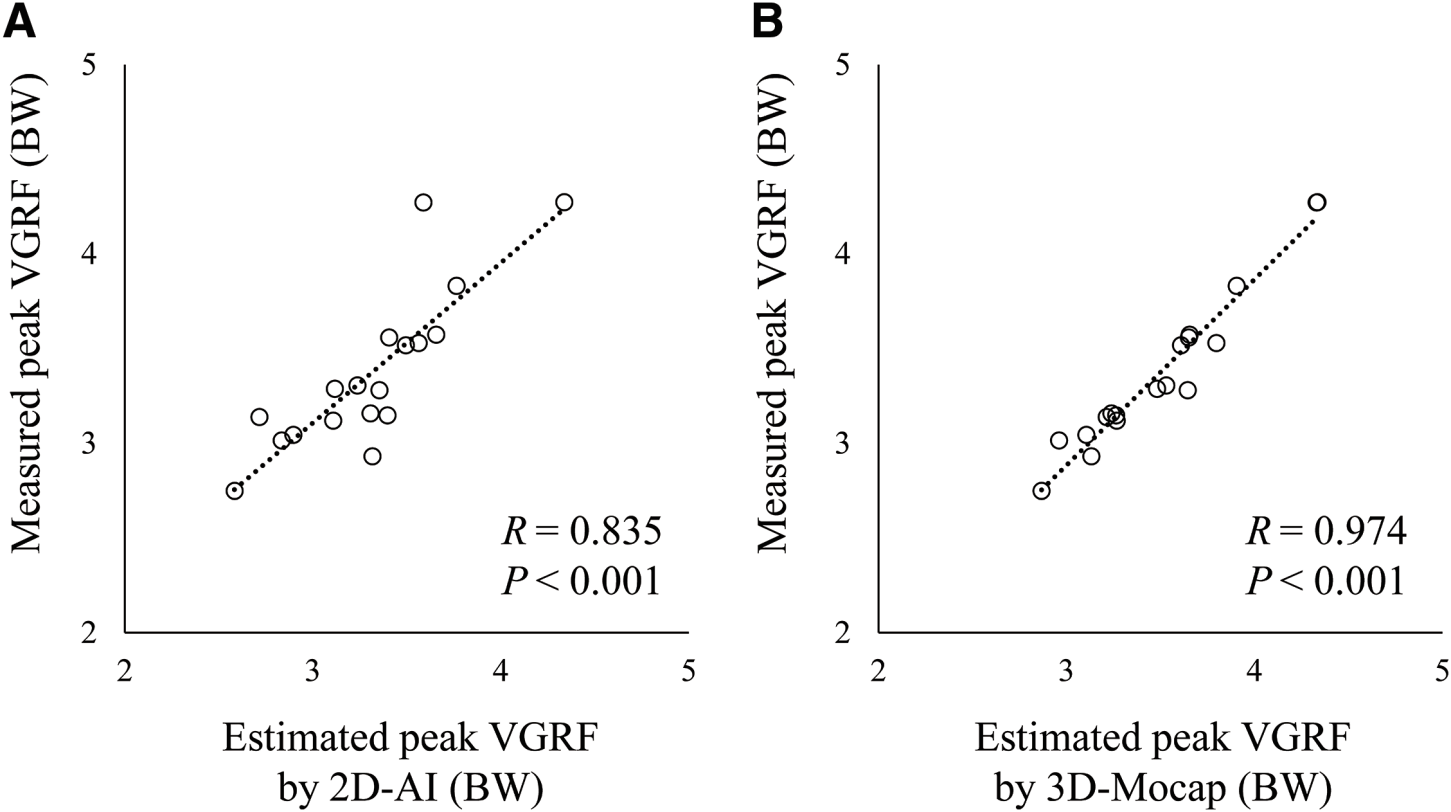
Correlation relationships of peak VGRF between measured and estimated by 2D-AI (A) and by 3D-Mocap (B) VGRF, vertical ground reaction force; BW, body weight; 2D, two-dimensional video image; AI, artificial intelligence; 3D-Mocap, three-dimensional optical motion capture

**Fig. 4.**
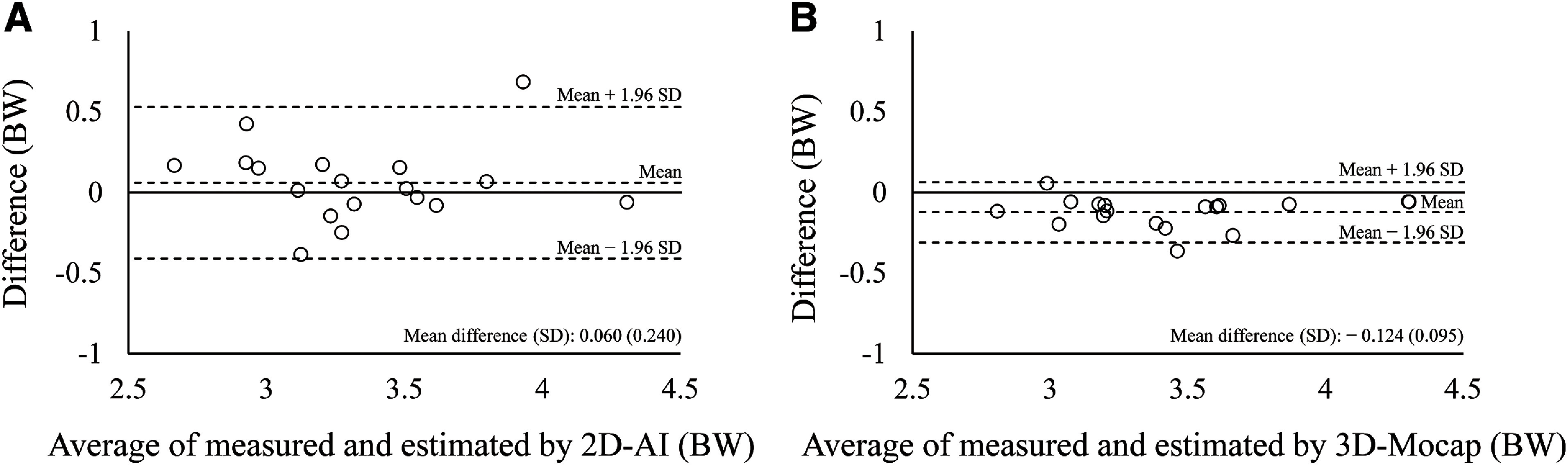
Bland–Altman plot demonstrating agreement between measured VGRF and estimated VGRF by 2D-AI (A) and 3D-Mocap (B) VGRF, vertical ground reaction force; BW, body weight; 2D, two-dimensional video image; AI, artificial intelligence; 3D-Mocap, three-dimensional optical motion capture

ICC (1, 3) of the peak VGRF (95% confidence interval [CI]) was 0.933 (0.854–0.973), 0.963 (0.920–0.985), and 0.928 (0.844–0.971) for the force plate measured, 2D-AI estimated and 3D-Mocap estimated VGRF, respectively. The peak VGRF was 3.37 BW (0.42 BW), 3.32 BW (0.42 BW), and 3.50 BW (0.42 BW) for the force plate measured, 2D-AI estimated and 3D-Mocap estimated VGRF, respectively. The measured VGRF demonstrated a substantial linear relationship with the VGRF estimated by 2D-AI, with a very large coefficient (*R* = 0.835, *P* <0.001), and with the VGRF estimated by 3D-Mocap, with an extremely large coefficient (*R* = 0.974, *P* <0.001) (Fig. 3). Bland–Altman analysis showed no fixed error (*P* = 0.307) or proportional error (*R* = 0.021, *P* = 0.934) for the VGRF estimated by 2D-AI (Fig. 4A). On the other hand, a significant fixed error was found for the VGRF estimated by 3D-Mocap (*P* <0.001) (Fig. 4B). There was no proportional error for estimated VGRF by 3D-Mocap (*R* = 0.032, *P* = 0.899). The AE of the estimated peak VGRF was 0.20 BW (0.16 BW) and 0.13 BW (0.09 BW) for 2D-AI and 3D-Mocap, respectively (*P* = 0.163). The RMSEs during the analyzed phase were 0.50 BW (0.12 BW) and 0.25 BW (0.13 BW) for the 2D-AI and 3D-Mocap estimations, respectively (*P* <0.001).

ICC (1, 3) of the time to peak VGRF (95% CI) was 0.920 (0.827–0.968), 0.705 (0.359–0.880), and 0.908 (0.801–0.63) for the force plate measured, 2D-AI estimated and 3D-Mocap estimated VGRF, respectively. The time to peak VGRF from IC was 71.1 ms (9.6 ms), 91.4 ms (6.5 ms), and 74.4 ms (12.5 ms) for the force plate measured, 2D-AI estimated, and 3D-Mocap estimated VGRF, respectively. The estimated time to peak was significantly later than the measured time to peak for both the 2D-AI and 3D-mocap (*P* <0.001 and *P* = 0.006, respectively).

## Discussion

The present study showed a significant linear relationship with a very large coefficient between the measured VGRF and the VGRF estimated by 2D-AI. In addition, the VGRF estimated by 2D-AI did not show a fixed or proportional error for the peak VGRF, and the AE was comparable to that estimated by 3D-Mocap. These results support the clinical application of assessing the VGRF during single-leg landings using 2D video images and pose estimation AI.

A strong linear relationship was found between the measured VGRF and VGRF estimated by 2D-AI (*R* = 0.835), which is comparable to those between the measured and estimated VGRFs during landing tasks using an accelerometer (*R* = 0.580–0.900)^35,36)^. In addition, no significant difference was found in the AE of the peak VGRF between the estimation using 2D-AI and that using 3D-Mocap. Bland–Altman analysis revealed no significant fixed bias or proportional error for the VGRF estimated by 2D-AI. The AE of the peak VGRF estimated by 2D-AI (0.20 BW) was comparable to the previously reported AE of the peak VGRF for running at 4.5 m/s (0.09–0.20 BW), acceleration of sprinting (0.33 BW) and decelerations of sprinting (0.78 BW) estimated using optical 3D motion capture^17,19)^. The present results indicate that the peak VGRF estimation using 2D video images and pose estimation AI has comparable accuracy to those using an optical 3D motion capture system and accelerometer^17,35,36)^.

The 2D-AI estimation was inferior to the 3D-Mocap estimation in several aspects. The Pearson’s correlation coefficient between the measured and estimated peak VGRF was smaller for the estimation by 2D-AI than for that by 3D-mocap. The RMSE was significantly larger for the estimation by 2D-AI than for that by 3D-Mocap. The difference in time to peak VGRF from the force plate measurement was also larger for 2D-AI estimation than for 3D-Mocap estimation. Several factors may contribute to the differences between the two estimation methods. First, errors in key point detection affect the estimation of COM position by 2D-AI. For VGRF estimation using a machine learning approach, pose estimation algorithms significantly affect the accuracy of VGRF estimation^37)^. Second, 2D video analysis may overestimate or underestimate the vertical displacement of the COM because this analysis cannot distinguish between vertical displacement and depth displacement. This limitation causes errors in the estimation of VGRF using 2D video.

The RMSEs during the analyzed phase were larger than the AE of the peak VGRF. The time to peak VGRF was also significantly different between the force plate measurement and both 2D-AI and 3D-Mocap estimation. From the comparison of the time curves, the post-peak decrease appeared to be different between the measured VGRF and the 2D-AI estimated VGRF. These results suggest that estimating the time-course change of VGRF from kinematics is inferior to estimating the peak VGRF, especially for 2D-AI estimation. Some approaches have been reported to reduce errors in estimating the VGRF from kinematics. As the present study showed that estimation errors were influenced by the cut-off frequency, filtering techniques may reduce these errors. In the 3D Mocap-based VGRF estimation, the individual cut-off frequency of the lowpass filter was set for each thigh and pelvis segment to reduce errors^17)^. Mass-spring-damper models are also used to estimate the VGRF during sports activities^38)^. These approaches may improve the impact force estimation but need some parameters such as stiffness and damping coefficients^38)^. A standard procedure to determine these coefficients has not been established, and the calculation is complex^38)^. These disadvantages of mass-spring-damper models limit clinical applications.

The peak VGRF is commonly evaluated in biomechanical assessment during landing tasks^11)^, and a limb asymmetry larger than 10% is considered as a clinically meaningful difference^39)^. The mean side-to-side difference in the peak VGRF during single-leg landing was reported to be 0.47 BW in patients after ACL reconstruction (ACLR)^40)^. Patients after ACLR who returned to their pre-injury competitive sports level showed a 0.35 BW larger peak VGRF than patients who did not return to their pre-injury sports level^12)^. Additionally, larger VGRF during landing is also associated with larger Achilles tendon force and patellofemoral joint stress^41,42)^. Therefore, the estimation using 2D video images and pose estimation AI may be a clinically useful method to detect a certain clinical difference in the peak VGRF during a single-leg landing. However, it should be noted that there was 0.2 BW of AE between the peak VGRF estimated by 2D-AI and that measured by a force plate. The present study used a commercial digital video camera. Recently, smartphones have also captured high-quality video footage at a sampling frequency of 200 Hz or higher. Therefore, the approach of this study may apply to telerehabilitation and sports injury prevention as well as clinical practice. Further studies are needed to verify the estimation of VGRF during landing using smartphone cameras.

Some limitations of this study should be acknowledged. First, we included only healthy male participants. Future studies should enroll patients, including those who are female, after ACLR. Second, we examined only the single-leg landing from a 30-cm height. Further study is needed to investigate other landing tasks and heights. Third, no additional correction for lens distortion was made outside of the capabilities of the camera. Additional lens distortion correction may improve the accuracy of estimation. Finally, the present study investigated the estimation of only VGRF. Large posterior ground reaction force is correlated to proximal anterior tibial shear force^43)^. Future studies should examine the estimation of anterior-posterior and medial-lateral GRF.

## Conclusions

The present study estimated the VGRF during a single-leg landing using a 2D video footage and pose estimation AI. The estimated VGRF was significantly correlated with the measured VGRF with a very large coefficient. The AE of the estimated peak VGRF was 0.20 BW and 6% relative to that of the measured peak VGRF, and no fixed bias or proportional error was found. There was no significant difference in the AE of the peak VGRF between the estimation using a 2D video image with pose estimation and that using a 3D motion capture system. The present findings indicate that VGRF estimation using 2D video images and pose estimation AI is a clinically useful method for assessing a single-leg landing, especially for peak value assessment.

## Acknowledgment

This work was supported by Japanese Society of Physical Therapy and JSPS KAKENHI (Grant Number: JP20K19317).

## Conflicts of Interest

The authors have no conflicts of interest to declare.
